# Clinical efficacy of Endostar continuous infusion combined with concurrent chemoradiotherapy in the treatment of oesophageal squamous cell carcinoma

**DOI:** 10.3389/fmed.2024.1463041

**Published:** 2024-10-23

**Authors:** Xinglong Du, Yuting Ji, Wenqiang Qin, Jie Wei

**Affiliations:** Department of Radiotherapy, The Affiliated Chuzhou Hospital of Anhui Medical University, Chuzhou, China

**Keywords:** Endostar, continuous infusion, concurrent chemoradiotherapy, oesophageal squamous cell carcinoma, clinical efficacy

## Abstract

**Objective:**

To evaluate the effectiveness and safety of concurrent chemoradiotherapy using Endostar continuous infusion for treating oesophageal squamous cell carcinoma (OSCC).

**Method:**

A total of 62 patients with oesophageal carcinoma were divided into three groups: the Endostar continuous infusion group (*n* = 27), the Endostar intravenous drip group (*n* = 21) and the concurrent chemoradiotherapy group (*n* = 14). All patients underwent oesophageal radiotherapy (56–60 Gy) alongside concurrent chemotherapy (4 mg of raltitrexed +100 mg of oxaliplatin, two cycles). In the Endostar continuous infusion group, 210 mg of Endostar was administered via infusion once every 3 weeks for 72 h, repeated for two cycles. The Endostar intravenous drip group received a dosage of 15 mg/day of Endostar, administered once daily for 14 days, repeated for two cycles. The objective response rate (ORR) (complete remission + partial remission), progression-free survival (PFS), 2-year overall survival (2y-OS) and adverse reactions were observed.

**Results:**

In the Endostar continuous infusion, intravenous drip and concurrent chemoradiotherapy groups, the ORR was 100, 95.2 and 78.6%, respectively (*p* < 0.05). There was a statistically significant difference between the continuous infusion and concurrent chemoradiotherapy groups (*p* < 0.05). However, there was no statistically significant difference between the continuous infusion and intravenous drip groups or the intravenous drip and concurrent chemoradiotherapy groups (*p* > 0.05). The continuous infusion and intravenous drip groups had higher PFS rates than the concurrent chemoradiotherapy group (*p* < 0.05). Regarding the 2y-OS rate, no statistically significant difference was observed among the three groups (*p* > 0.05). Furthermore, there was no statistically significant difference in adverse reactions among the groups (*p* > 0.05).

**Conclusion:**

Concurrent chemotherapy based on endostatin is effective and safe in the treatment of OSCC. Continuous 3-day Endostar infusion treatment can significantly enhance both short-and long-term therapy efficacy in patients while maintaining a high level of safety.

## 1 Introduction

Concurrent chemoradiotherapy has emerged as the primary approach for the radical treatment of oesophageal carcinoma, notably extending survival time (1). Raltitrexed, which is capable of inhibiting thymidylate synthase with minimal side effects, is increasingly incorporated into concurrent chemotherapy regimens for oesophageal cancer, yielding enhanced efficacy (2, 3). Currently, the standard approach to treating oesophageal cancer typically involves a combination of concurrent chemoradiotherapy alongside immunotherapy and targeted anti-angiogenesis medications (4, 5). Angiogenesis is pivotal in the progression, invasion and metastasis of malignant solid tumors. Consequently, anti-angiogenesis therapy holds significant promise in treating various tumor types. As a result, there is a growing focus on incorporating anti-vascular drugs into the comprehensive management of oesophageal cancer. Approximately 95% of cases of oesophageal carcinoma in China are squamous cell carcinomas (6). Since patients with oesophageal carcinoma have varying degrees of dysphagia, most small-molecule anti-angiogenic drugs cannot be taken orally.

Endostar is an innovative recombinant human endostatin injection, a new generation of biologics that has shown significant potential in the field of cancer treatment. Its therapeutic mechanism primarily relies on the inhibition of tumor angiogenesis (7), a critical component of tumor growth and metastasis. By specifically targeting vascular endothelial growth factor (VEGF), Endostar blocks the binding of VEGF to its receptors, thereby inhibiting the proliferation of vascular endothelial cells and effectively reducing the formation of new blood vessels (8). This process not only cuts off the tumor’s supply of nutrients, limiting its growth, but also reduces the risk of tumor cells migrating to other parts of the body through blood vessels to form metastatic tumors. Additionally, it can work synergistically with other treatment modalities, such as chemotherapy and radiotherapy, to enhance their efficacy. Clinical studies have shown promising results for Endostar in various cancer types, including lung cancer, nasopharyngeal carcinoma and gastric cancer (9–11). Pan-target anti-angiogenesis modulates the dynamic equilibrium of angiogenesis within the tumor microenvironment, facilitating its normalization. This normalization enhances chemotherapy sensitivity, consequently impeding tumor growth and metastasis, leading to objectively prolonged patient survival time (12, 13). When combined with chemoradiotherapy, Endostar has demonstrated improved efficacy in treating lung cancer (14) and nasopharyngeal cancer (15), as well as other tumor types, alleviating concerns about bleeding associated with squamous cell carcinoma. Moreover, it has relatively few side effects and relatively high safety levels (16, 17). Endostar has a half-life of 8–12 h. The traditional route of administration of Endostar is 7.5 mg/m^2^ via intravenous drip for 3–4 h per day for 14 consecutive days. However, the traditional use of intravenous drip causes large fluctuations in drug concentration and limits the compliance of patients with long-term administration of Endostar. Studies have shown that the anti-tumor effect of Endostar is time-and concentration-dependent, and its anti-tumor effect will increase with the extension of medication time and the increase of blood drug concentration within a certain range. Kisker (18) found that after a single intraperitoneal injection of Endostar to mice, the drug in the tumor tissue was quickly cleared within 2 h. Moreover, continuous administration through a micro-osmotic pump can keep the blood drug concentration stable for a long time, and the same anti-tumor effect can be obtained with one-eighth of the dose of a single injection. This may be due to the continuous intravenous pumping of Endostar, which allows the drug solution to be continuously and evenly infused, prolongs the infusion time and can maintain a stable blood drug concentration, such that the drug can continue to act on the endothelial cells of the new blood vessels, thereby achieving a better anti-tumor effect. Clinical studies have indicated that the continuous intravenous infusion of recombinant human endostatin yields superior outcomes compared with intravenous drip treatment (19–21), without an associated increase in side effects.

In the treatment of locally advanced oesophageal squamous cell carcinoma (LA-OSCC), concurrent chemoradiotherapy has become the standard treatment plan. With the rise of immunotherapy, researchers have begun to explore new combined treatment plans to improve therapeutic outcomes. Recently, a single-center, open-label phase II study demonstrated the potential of Endostar in combination with envafolimab (a PD-L1 checkpoint inhibitor) and concurrent chemoradiotherapy for the treatment of LA-OSCC. In this study, patients received 50.4 Gy of radiotherapy, chemotherapy with paclitaxel liposome and carboplatin, and treatments with Endostar and envafolimab. Preliminary results showed that this combined treatment plan has good tolerability and controllable toxicity, and all patients experienced a reduction in target lesion size, with an objective response rate (ORR) of 100% and an endoscopic complete remission rate of 88.9%. These promising results provide strong support for the ongoing phase II study and offer new therapeutic hope for patients with LA-OSCC (22).

However, there has been no study reporting the efficacy of Endostar continuous infusion combined with raltitrexed-based concurrent chemoradiotherapy in the treatment of OSCC. Therefore, this study reports the efficacy and safety of Endostar continuous infusion combined with concurrent chemoradiotherapy in treating this disease.

## 2 Information and methodology

### 2.1 Inclusion criteria

The inclusion criteria for patients were as follows: (1) aged 45–75 years; (2) pathological confirmation of squamous cell carcinoma; (3) ability to tolerate concurrent chemoradiotherapy; (4) no previous history of bleeding and concomitant diseases; (5) routine physical examination, blood routine, liver function, kidney function and other auxiliary examinations not contraindicated by radiotherapy and chemotherapy; and (5) informed consent obtained and signed prior to treatment. The exclusion criteria were as follows: (1) severe underlying diseases and unable to tolerate concurrent chemotherapy; (2) a history of significant peptic ulcer; and (3) a history of previous bleeding. The general information collected included age, location, differentiation, tumor stage and angiographic classification. Tumor staging was performed using the Union for International Cancer Control (UICC) guidelines (2002 edition).

### 2.2 General information

A total of 62 patients (38 men and 24 women) with OSCC admitted to the department of radiotherapy between February 2017 and December 2020 were randomly divided into three groups. The patients’ ages ranged from 45 to 75 years, with a median age of 63.0 and an average age of 63.60 ± 7.34. The distribution skewness was −0.283 ± 0.304, which conformed to a normal distribution. The ages provided were those at the time of the patient’s initial radiation treatment. In addition, the time between the first diagnosis and first radiation treatment for all patients did not exceed 3 months. Patients were randomly assigned to either the Endostar continuous infusion group, the Endostar intravenous drip group or the concurrent chemoradiotherapy group based on the day of the week of admission. Patients admitted on Monday and Thursday were assigned to the Endostar continuous infusion group, those admitted on Tuesday and Friday were assigned to the Endostar intravenous drip group and those admitted on Wednesday and Saturday were assigned to the concurrent chemoradiotherapy group. None of the patients assigned to these groups had received any anti-tumor treatment prior to the therapy. Ethical approval for this study was obtained from the institutional ethical committee. The treatment choice for the randomly assigned patients was independent of both gender and age. The general treatments administered to the enrolled patients are detailed in Table 1.

**Table 1:** Patient general information.

	ENDOSTAR continuous infusion group (*n* = 27)	ENDOSTAR intravenous drip group (*n* = 21)	Concurrent chemoradiotherapy group (*n* = 14)	*χ*^2^/*F* value	*p* value
Male	16 (59%)	13 (62%)	9 (64%)	0.103	*p* = 0.950
Female	11 (41%)	8 (38%)	5 (36%)		
Age	63.22 ± 1.01	64.09 ± 1.12	63.57 ± 2.07	1.043	*p* = 0.439
Smoking					
Yes	18 (67%)	14 (67%)	10 (71%)	0.112	*p* = 0.945
No	9 (33%)	7 (33%)	4 (29%)		
Alcohol drinking					
Yes	20 (74%)	15 (71%)	11 (79%)	0.224	*p* = 0.894
No	7 (26%)	6 (29%)	3 (21%)		
TNM stage					
Stage II	4 (15%)	4 (19%)	3 (21%)	0.369	*p* = 0.985
Stage III	17 (63%)	13 (62%)	8 (57%)		
Stage IV	6 (22%)	4 (19%)	3 (22%)		
Highly differentiated	8 (30%)	6 (29%)	4 (29%)	0.321	*p* = 0.988
Intermediate differentiation	15 (56%)	11 (52%)	7 (50%)		
Low differentiation	4 (14%)	4 (19%)	3 (21%)		
≤6 cm	19 (70%)	16 (76%)	10 (71%)	0.213	*p* = 0.899
>6 cm	8 (30%)	5 (24%)	4 (29%)		
Medullary type	20 (74%)	18 (86%)	10 (71%)	7.465	*p* = 0.280
Ulcer type	2 (7%)	0 (0%)	1 (7%)		
Narrow type	2 (7%)	1 (5%)	1 (7%)		
Intracavity type	3 (12%)	2 (9%)	2 (14%)		

### 2.3 Methods

All patients underwent radiotherapy alongside concurrent chemotherapy based on raltitrexed. The concurrent chemoradiotherapy group received radiotherapy combined with concurrent chemotherapy alone. The Endostar continuous infusion group received Endostar administered via a pump in addition to concurrent chemoradiotherapy. The Endostar intravenous drip group received Endostar administered intravenously via a drip in addition to concurrent chemoradiotherapy.

#### 2.3.1 Radiotherapy

The patient’s position was fixed with thermoplastic film, and enhanced computed tomography (CT) simulation localisation was performed. The image was uploaded to the Pinnacle intensity-modulated radiation therapy (IMRT) system. The target area was delineated based on the CT scan image, gastroscopy and oesophagography. Gross tumor volume (GTV) delineated the focus of oesophageal carcinoma, whereas nodal GTV (GTVn) included enhanced CT images of metastatic lymph nodes. Clinical target volume (CTV) encompassed both GTV and GTVn, extending the upper and lower ends of GTVn outward by 0.5 cm and incorporating the corresponding lymph node drainage area with a 0.6-cm margin at each end. Planning gross tumor volume (PGTV) comprised GTV and GTVn, with the upper and lower ends of GTV extended by 3–5 cm and the upper and lower ends of GTVn extended outward by 0.5 cm, along with an additional extension of 0.5 cm on the anterior, posterior, left and right sides. Planning target volume underwent a three-dimensional (3D) CTV expansion of 0.5 cm, followed by adjustments to organs at risk, such as large blood vessels and vertebral bodies. In terms of prescription dose, 95% of PGTV received 50.4–60 Gy in 1.8–2 Gy fractions over 28–30 fractions, and 95% of PGTV received 59.92 Gy in 2.14 Gy fractions over 28 fractions. The maximum dose to organs at risk was as follows: spinal cord, maximum <40 Gy; lungs, V20 < 28%; and heart, V40 < 30%. Routine segmentation occurred once per day, five times per week. Following the completion of the radiotherapy plan, the deputy chief physician confirmed and verified the treatment dose before execution. The treatment was administered using a Siemens accelerator (6MV-X IMRT; Siemens AG, Munich, Germany).

#### 2.3.2 Chemotherapy

All patients received concurrent chemotherapy based on raltitrexed. The first week of radiotherapy was followed by 4 mg of raltitrexed +100 mg of oxaliplatin and was repeated for 3 weeks, completing two cycles.

#### 2.3.3 Nutritional support and other treatments

Raltitrexed (Nanjing Zhengda Tianqing Pharmaceutical Co., Ltd., Nanjing, China) was administered at a dose of 2 mg per vial, whereas oxaliplatin (Jiangsu Hengrui Pharmaceutical Co., Ltd., China; Jinan Qilu Pharmaceutical Co., Ltd., China) was given at a dose of 100 mg per vial.

#### 2.3.4 Targeted therapy

The Endostar continuous infusion group received 210 mg of endostatin +102 mL of normal saline (NS) at the beginning of the first day of radiotherapy for 72 h once every 3 weeks, totalling two cycles. The Endostar intravenous drip group received 15 mg of endostatin +500 mL of NS intravenously for 3 h from the first day of radiotherapy once daily for 14 consecutive days, followed by 7 days of rest, completing two cycles. Endostar was used at a concentration of 15 mg/mL (First Sound Pharmaceutical, Jiangsu, China).

### 2.4 Observation index and judging standard

The TNM staging was performed for all patients using the UICC guidelines (2002 edition) staging system. Enhanced CT and oesophagography were performed within 1–2 months of radiotherapy. Wan et al. (23) initially proposed the efficacy standard for oesophageal carcinoma, categorizing oesophageal lesions as either in complete remission (CR), in partial remission (PR), stable (SD) or progressive (PD) based on oesophagography. Mediastinal lymph nodes were identified as complete response (CR), partial response (PR), SD or PD using chest CT according to the World Health Organization’s RECIST1.1 criteria. The overall therapeutic effect was judged in combination with the changes in oesophageal lesions and mediastinal lymph nodes. If the results of the two tests were not completely consistent, this study adopted the lowest therapeutic effect as the comprehensive therapeutic effect result (see Table 2). The ORR was defined as including the comprehensive therapeutic effect results of CR and PR. For long-term effects, the median progression-free survival (mPFS) and the median 2-year overall survival (median 2y-OS) were observed. The mPFS was defined as the point prior to which 50% of the patients had not shown disease progression after the start of radiation therapy. The median 2y-OS was defined as the number of patients who survived at least 2 years after the start of radiation treatment. Adverse reactions to the chemotherapy drugs were assessed according to the Common Terminology Criteria for Adverse Events (CTCAE) 4.0 standard (May 2009), and the Radiation Therapy Oncology Group’s (RTOG) acute radiation injury grading standard (24) was used to assess the side effects of radiation therapy.

**Table 2:** Comparison of short-term efficacy among three groups.

	ENDOSTAR continuous infusion group (*N* = 27)	ENDOSTAR intravenous drip group (*N* = 21)	Concurrent chemotherapy group (*N* = 14)
CR	9/27 (33.3%)	6/21 (28.6%)	3/14 (21.4%)
PR	18/27 (66.7%)	14/21 (66.7%)	8/14 (57.1%)
SD	0/27 (0)	1/21 (4.8%)	3/14 (21.4%)
ORR (%)	100	95.2	78.6

The *p*- value for comparison between the 3 groups was *p* = 0.031 (*p* < 0.05). Comparisons between subgroups require a corrected P value of P′. P′ ENDOSTAR continuous infusion group/Concurrent chemotherapy group = 0.014 (*p*′<0.017), *P*′ ENDOSTAR continuous infusion group / ENDOSTAR intravenous drip group = 0.508 (*p*′>0.017), *P*′ ENDOSTAR intravenous drip group/Concurrent chemotherapy group = 0.143 (*p*′ > 0.017).

The ORR included the CR and PR, and the lowest curative effect was taken as the standard. Progression-free survival and 2y-OS were observed during telephone and outpatient follow-ups of 24 months. The side effects of radiotherapy were assessed according to the May 2009 CTCAE 4.0 criteria for adverse reactions to chemotherapy and the RTOG’s acute radiation injury grading criteria (24).

### 2.5 Follow-up

Telephone and outpatient follow-ups were conducted for all patients following radiotherapy and chemotherapy. Follow-up examinations were performed every 3 months in the first year and every 4–5 months in the second year. The follow-up indices included alleviation of eating obstruction, oesophagography and chest CT imaging. All patients were followed up for 24 months, and the deadline for follow-up was December 2022. One patient died from an accidental incident and two died from heart disease; none of the patients were lost to follow-up, with a 100% follow-up rate.

### 2.6 Statistical methods

All results were analyzed using the SPSS 25.0 statistical software. The mean ± standard error was used to summarize the measurement data, and the ORR was expressed as a percentage. Patient data and adverse drug reactions were analyzed using the chi-square (*χ*^2^) test. The ORR was assessed using analysis of variance with a random block design. The PFS and 2y-OS were evaluated for differences in survival using the Breslow test. Kaplan–Meier survival curves were subsequently constructed, with a significance level (*α*) set at 0.05.

## 3 Results

### 3.1 Comparison of short-term efficacy

The CR, PR and SD were 9 (33.30%), 18 (66.7%) and 0 (0%), respectively, in the Endostar continuous infusion group. The CR, PR and SD were 6 (28.6%), 14 (66.7%) and 1 (4.8%), respectively, in the Endostar intravenous drip group. In the concurrent chemoradiotherapy group, the CR, PR and SD were 3 (21.4%), 8 (57.1%) and 3 (21.4%), respectively. The ORRs of the three groups were 100, 95.2 and 78.6%, respectively, and the difference among the three groups was statistically significant (*p* = 0.031). The ORR of the Endostar continuous infusion group (100%) was significantly higher than that of the concurrent chemoradiotherapy group (78.2%) (*p* = 0.014). There was no significant difference between the continuous infusion and intravenous drip groups or between the intravenous drip and concurrent chemoradiotherapy groups (*p* > 0.05; see Table 2).

### 3.2 Survival analysis

#### 3.2.1 Progression-free survival

**Figure 1:**
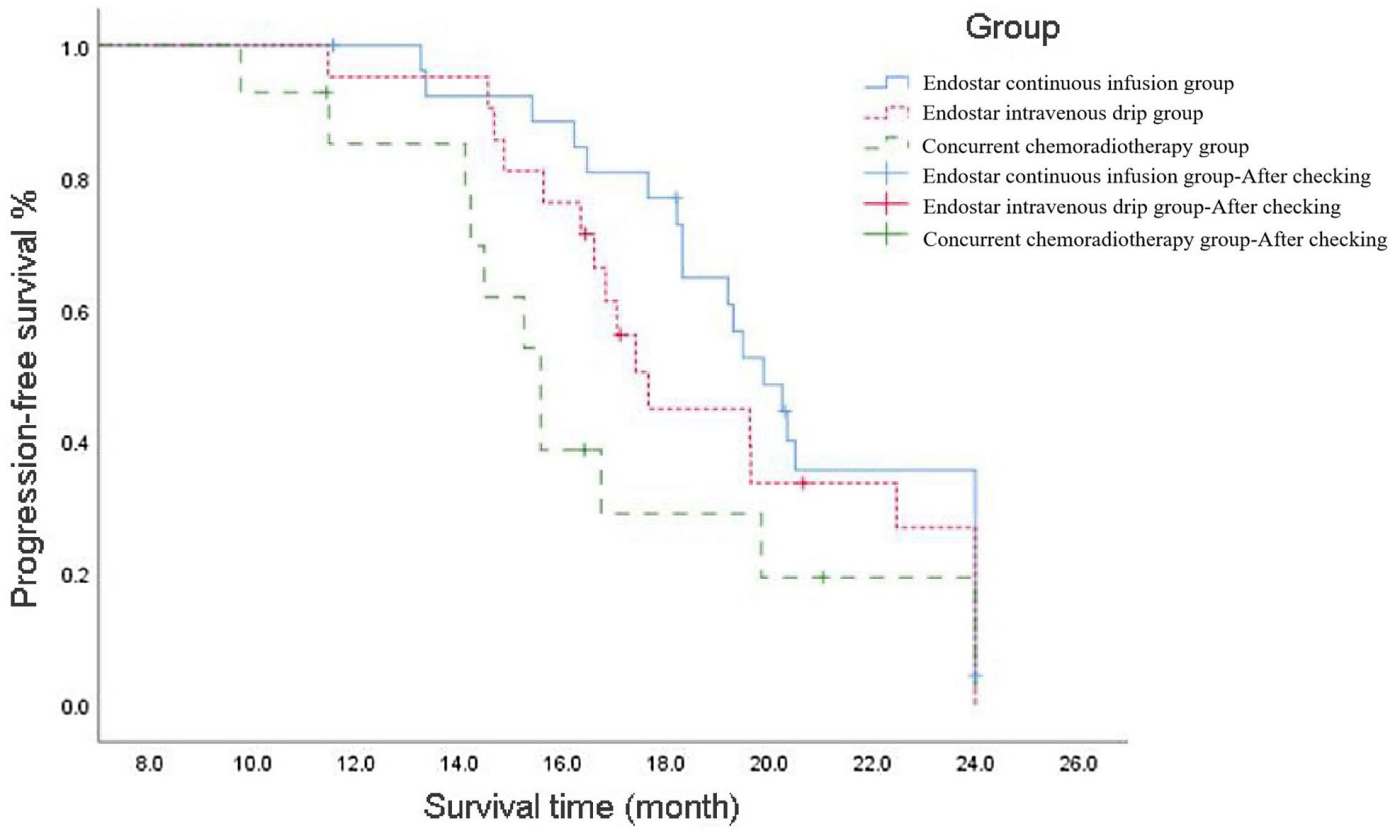
Progression-free survival curve of ENDOSTAR continuous infusion group, ENDOSTAR intravenous drip group and concurrent chemotherapy group. The curves depict the rates at which patients in each group maintained disease progression-free status over time since the commencement of treatment.

The mPFS was 19.9 [95% confidence interval (CI): 18.39–21.42] months in the Endostar continuous infusion group, 17.7 (95% CI: 16.46–18.87) months in the Endostar intravenous drip group and 15.6 (95% CI: 14.3–16.84) months in the concurrent chemoradiotherapy group, and the difference was statistically significant (*p* = 0.003). Following correction using the Bonferroni method, the intergroup significance level was set at *α* = 0.017. Further analysis between the two groups revealed a statistical difference between the Endostar continuous infusion group and the concurrent chemotherapy group (*p* < 0.017), but there was no statistically significant difference between the intravenous drip group and the concurrent chemotherapy group or between the continuous infusion group and the intravenous drip group (*p* > 0.017; see Figure 1).

#### 3.2.2 Two-year overall survival

**Figure 2:**
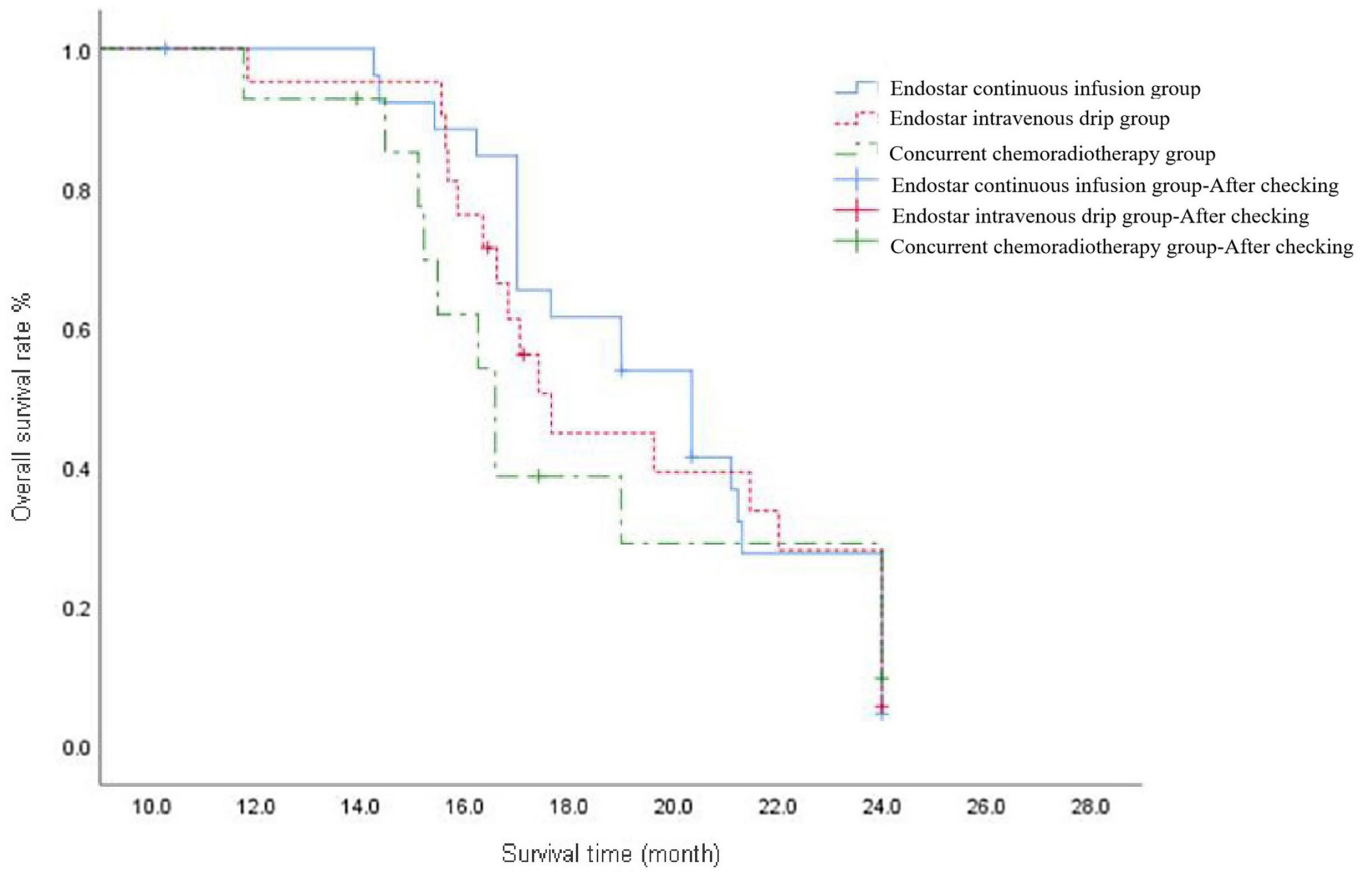
The overall survival curve of ENDOSTAR continuous infusion group, ENDOSTAR intravenous drip group and concurrent chemotherapy group. The curves reflect the survival conditions of patients in each group from the start of treatment until the end of follow-up.

The median 2y-OS response rate was 20.35 (95% CI: 17.77–22.93), 17.7 (95% CI: 16.46–18.87) and 16.6 (95% CI: 15.32–17.83) months in the Endostar continuous infusion, Endostar intravenous drip and concurrent chemotherapy groups, respectively, and the difference was not statistically significant (*p* = 0.090). Following correction with the Bonferroni method, the intergroup significance level was set at *α* = 0.017. Further analysis found no statistical difference between the Endostar continuous infusion and concurrent chemotherapy groups, between the intravenous drip and concurrent chemotherapy groups or between the Endostar continuous infusion and intravenous drip groups (*p* > 0.017; see Figure 2).

### 3.3 Adverse reactions

The major adverse events were radiation oesophagitis (grades I–II and III–IV), decreased neutrophil counts and fatigue, and neutropenia was managed with CSF3. Thrombocytopenia treatment with interleukin 11 and thrombopoietin was successfully completed. During that period, one case of III–IV grade hypertension was relieved by adjusting the dose of antihypertensive drugs, and the treatment was resumed. One case in each group of grade I–II haematemesis was found in all three groups, and no radiation pneumonitis occurred in any of the three groups (see Table 3).

**Table 3:** The adverse reactions of 62 cases of esophageal carcinoma with different therapeutic schemes.

Adverse reactions	ENDOSTAR continuous infusion Group		ENDOSTAR intravenous drip group		Concurrent chemoradiotherapy group		*X*^2^_value_	*p*- value
	I-II	III-IV	I-II	III-IV	I-II	III-IV		
Thrombocytopenia	7	2	6	2	4	1	0.017	0.895
Less neutrophil	14	3	11	3	10	2	0.021	0.796
Vomiting blood	1	0	1	0	1	0	0	1.000
High blood pressure	7	1	8	0	1	0	−0.111	0.288
Radiation esophagitis	24	3	16	5	12	2	0.083	0.241
Hoarse voice	4	0	2	0	0	0	−0.286	0.439
Hard to breathe	1	0	1	0	1	0	0	1.000
Fatigue	23	4	18	3	12	2	−0.004	0.958
Nausea/vomiting	17	2	18	1	10	2	−0.032	0.589

## 4 Discussion

This study concluded that Endostar continuous infusion combined with concurrent chemoradiotherapy can improve the ORR of oesophageal carcinoma. Inhibition of the VEGF-related signaling pathway may promote the efficacy of radiotherapy for this type of carcinoma (25). Fan et al. (26) found that Endostar combined with concurrent chemoradiotherapy increased the ORR of OSCC (*p* = 0.026). The ORR of concurrent chemoradiotherapy with Endostar and tetrandrine for recurrent oesophageal cancer was 66.7%, significantly surpassing that of the control group (39.3%) (*p* < 0.05) (27). In a study by Liang (28), which included 62 patients with advanced ovarian cancer, the findings indicated that there was no difference in the overall response rate between the paclitaxel/platinum plus Endostar intravenous infusion and intravenous infusion regimens (*p* > 0.05). However, some clinical studies have revealed that the ORR of Endostar continuous infusion is better than that of intravenous infusion. Zhang et al. (21) reported 100 cases of advanced non-small cell lung carcinoma cancer (NSCLC) treated with Endostar continuous infusion and infusion combined with platinum-containing dual-drug chemotherapy. The results demonstrated that the intravenous infusion of ORR (*p* = 0.026) and disease-control rate (*p* = 0.017) were superior to intravenous infusion. In the present study, the ORR was 95.2 and 78.2% in the Endostar intravenous drip and concurrent chemoradiotherapy groups, respectively, and there was no statistically significant difference (*p* > 0.05). These results differ from those of existing clinical studies. The absence of a statistical difference between the two groups may be attributed to an increased likelihood of random error due to the smaller number of patients in both groups. However, there was a significant difference between the Endostar continuous infusion and concurrent chemotherapy groups (*p* = 0.036). It may be advantageous to explore cumulative differences between the Endostar continuous infusion and Endostar intravenous drip groups, as well as between the intravenous and concurrent chemoradiotherapy groups.

This study found that there was a significant difference in mPFS between the three groups, and subgroup analysis showed that the difference in PFS between the Endostar continuous infusion group and the concurrent chemotherapy group was significant. It is suggested that Endostar combined with radiotherapy and chemotherapy can enhance the PFS of patients, with continuous infusion potentially offering more pronounced benefits. A retrospective analysis suggested that concurrent chemoradiotherapy combined with Endostar injection can improve the 3-year PFS rate of patients with locally advanced nasopharyngeal carcinoma (29). Another study suggested that the PFS of patients with nasopharyngeal cancer treated with concurrent chemoradiotherapy plus Endostar was significantly longer than that of concurrent chemoradiotherapy by approximately 4 months (30). However, in the present study, there was no significant difference between the Endostar continuous infusion group and the Endostar intravenous drip group, or between the Endostar intravenous drip group and the concurrent chemotherapy group, which is different from the results of published studies. A previous study suggested that continuous infusion of Endostar combined with chemotherapy may decrease the risk of locally advanced disease compared with intravenous drip, and the mPFS of NSCLC increased from 4.4 to 8.0 months (*p* = 0.019) (16). Xu et al. found that the PFS of 40 patients with intermediate-to-advanced NSCLC treated by the two routes of Endostar combined with a gemcitabine/cisplatin (GP) regimen was 7.5 and 5.9 months, respectively (*p* < 0.05) (31). A real-world analysis assessing the effectiveness of Endostar combined with chemotherapy in treating 54 cases of non-driver gene mutation NSCLC revealed that the mPFS of 7-day continuous infusion was superior to that of 14-day intravenous drip (6 vs. 4.5 months) (32).

The difference in our results can be attributed to the study’s design, which involved comparing three groups using two subgroups. This contrasts with other clinical studies, which typically compare only two groups. Furthermore, this study’s statistical test employed *p* < 0.017 as the threshold for test significance, whereas other studies typically used *p* < 0.05 as the criterion for significance. Moreover, the limited number of cases and short follow-up time may have introduced experimental bias.

This study suggests that whether or not Endostar is used, and irrespective of whether it is administered via continuous infusion or intravenous drip, there were no benefits in terms of the 2y-OS (*p* = 0.196). Moreover, the study did not demonstrate that the use of Endostar in combination with chemoradiotherapy can significantly improve long-term survival rates compared with chemoradiotherapy alone. Endostar also showed no significant benefit in the 2y-OS of patients with other tumors. The OS of recombinant human endostatin combined with a GP regimen and GP regimen alone was 12.7 and 12.3 months in 40 patients with advanced NSCLC, respectively (*p* > 0.05) (31). No prolonged OS was seen in a real-world study of the efficacy of chemotherapy combined with recombinant human endostatin following 7-day continuous infusion and 14-day intravenous infusion in 54 patients with NSCLC (*p* = 0.111) (32).

In the present study, radiation-induced oesophagitis, neutrophil levels, fatigue and vomiting were the primary adverse events. These adverse reactions (graded >3) occurred in 16.0% (10/62), 14.5% (9/62), 12.9% (8/62) and 8.1% (5/62) of patients, respectively. No oesophageal perforation or severe haematemesis occurred. The study assessed whether Endostar was used in combination and whether the drug was administered continuously over 3 days or infused intravenously over 14 days without causing life-threatening side effects. The results showed that Endostar is safe and reliable. Additionally, Endostar can be considered safe and manageable in other cancer combinations. A 3-day regimen of continuous intravenous infusion of recombinant human endostatin combined with chemotherapy was found to have a manageable adverse effect and an acceptable safety profile in 127 patients with solid tumors (33). A phase II study of Endal plus etoposide/cisplatin in 22 patients with small-cell carcinoma found that all patients tolerated the treatment. The main adverse reactions were myelosuppression, proteinuria, nausea and vomiting, and the incidence of grade 3 and 4 adverse reactions was 7.2%. No treatment-related deaths occurred (34). Endostar combined with chemoradiotherapy can further expand the benefits of treating the population with OSCC and, for more extensive cases, improve effective and safe treatment opportunities.

This study provides important insights in the field of concurrent chemoradiotherapy for OSCC. Based on this study, the use of Endostar combined with chemotherapy and radiotherapy for OSCC may have a range of potential long-term effects on patients. First, we discovered that combining Endostar with chemotherapy and radiotherapy yields notable efficacy and safety in treating this carcinoma type. Specifically, continuous infusion of Endostar notably enhances ORRs and PFS rates, offering fresh therapeutic avenues for clinical practice. Although no statistically significant difference in 2y-OS was found in this study, the improved treatment effect may help prolong patient survival. This result may be limited by sample size or follow-up time. Improved treatment efficacy and longer survival may reduce patients’ symptoms and complications, thereby improving their quality of life. Second, this study emphasizes the importance of personalized treatment approaches. By comparing various regimens, it was found that continuous Endostar infusion outperforms intravenous administration and simple chemoradiotherapy. This highlights the need to tailor treatments based on individual patient characteristics and pathology, ultimately improving treatment outcomes and survival rates. In addition, as an anti-angiogenic drug, Endostar may help reduce tumor recurrence and metastasis by inhibiting tumor angiogenesis, providing patients with longer-term disease control. However, although the side effects of Endostar were controllable in this study, the unknown long-term side effects that may be caused by long-term use need to be managed through continuous monitoring and evaluation. This study highlights the need for improved treatment options. Finally, this study may provide new directions for future research on Endostar and other anti-angiogenic drugs in OSCC, and even other types of cancer, to explore their long-term effects and optimal use strategies.

Despite our progress, limitations persist, including sample size constraints and short follow-up periods. Therefore, future research should prioritize enlarging the sample size, and further research involving more diverse populations and extended follow-up durations is needed to validate our findings and explore novel therapeutic strategies and drugs.

In summary, Endostar combined with raltitrexed-based concurrent chemoradiotherapy is safe and effective for the treatment of OSCC. Furthermore, 3D continuous infusion can improve the ORR and PFS rate. A 3-day continuous infusion of Endostar constitutes a short treatment duration, associated with mild adverse reactions, meaning it may offer an effective and safe approach to treating OSCC.

## Data Availability

The original contributions presented in the study are included in the article/supplementary material, further inquiries can be directed to the corresponding authors.
